# Modeling hypothermia induced effects for the heterogeneous ventricular tissue from cellular level to the impact on the ECG

**DOI:** 10.1371/journal.pone.0182979

**Published:** 2017-08-16

**Authors:** Roland Kienast, Michael Handler, Markus Stöger, Daniel Baumgarten, Friedrich Hanser, Christian Baumgartner

**Affiliations:** 1 Institute of Electrical and Biomedical Engineering, UMIT–University for Health Sciences, Medical Informatics and Technology, Hall, Tyrol, Austria; 2 Institute of Biomedical Engineering and Informatics, Technische Universität Ilmenau, Ilmenau, Germany; 3 Institute of Health Care Engineering with European Testing Center of Medical Devices, Graz University of Technology, Graz, Austria; University of Minnesota, UNITED STATES

## Abstract

Hypothermia has a profound impact on the electrophysiological mechanisms of the heart. Experimental investigations provide a better understanding of electrophysiological alterations associated with cooling. However, there is a lack of computer models suitable for simulating the effects of hypothermia in cardio-electrophysiology. In this work, we propose a model that describes the cooling-induced electrophysiological alterations in ventricular tissue in a temperature range from 27°C to 37°C. To model the electrophysiological conditions in a 3D left ventricular tissue block it was essential to consider the following anatomical and physiological parameters in the model: the different cell types (endocardial, M, epicardial), the heterogeneous conductivities in longitudinal, transversal and transmural direction depending on the prevailing temperature, the distinct fiber orientations and the transmural repolarization sequences. Cooling-induced alterations on the morphology of the action potential (AP) of single myocardial cells thereby are described by an extension of the selected Bueno-Orovio model for human ventricular tissue using Q10 temperature coefficients. To evaluate alterations on tissue level, the corresponding pseudo electrocardiogram (pECG) was calculated. Simulations show that cooling-induced AP and pECG-related parameters, i.e. AP duration, morphology of the notch of epicardial AP, maximum AP upstroke velocity, AP rise time, QT interval, QRS duration and J wave formation are in good accordance with literature and our experimental data. The proposed model enables us to further enhance our knowledge of cooling-induced electrophysiological alterations from cellular to tissue level in the heart and may help to better understand electrophysiological mechanisms, e.g. in arrhythmias, during hypothermia.

## Introduction

Hypothermia has a relevant impact on physiological regulatory mechanisms in the pulmonary, neurologic, hemostatic and cardiovascular system [1]. In a clinical setting, targeted temperature management (TTM) previously known as therapeutic hypothermia has become an important method of treatment to improve the neurological outcome, yielding a significant increase in long-term survival after cardiac arrest [2–4]. Deflecting the attention on the heart, hypothermia-induced electrophysiological changes can be observed through morphologic variances in the electrocardiogram (ECG) recording [5,6]. These variances originate at the cellular level and are primarily caused by alterations in ion channel dynamics. Most of these alterations are primarily attributed to electrophysiological changes within the ventricles. A closer look at the left ventricular cell structure reveals three different myocardial cell types: endocardial, midmyocardial (M), and epicardial cells with heterogeneous electrophysiological properties, showing distinct transmembrane potential morphologies [7,8]. Various experiments in mammalian ventricular wedge models have advanced our knowledge on the relationship between the effects of this electric heterogeneity on transmembrane potential in distinct cell types and its contribution to the morphology of registered ECG signals by simultaneous recording of transmembrane APs and pseudo-ECG (pECG). Utilizing this technique at hypothermic conditions led to the following primary findings:

On a cellular level hypothermia induces a prolongation of the action potential duration (APD) of myocardial cells, resulting in a change of the T-wave morphology and a prolongation of the QT interval in the ECG. A decrease in electrical conduction between connected cells was found due to a reduction in gap junction conductivity, thus resulting in a reduction in conduction velocity (CV) [9]. In ECG, this slowed CV is reflected as a prolongation in all electrocardiographic intervals. In ventricular tissue this effect especially leads to a prolonged QRS interval [10]. The prominent, cooling-induced formation of the J-wave is based on a distinctive notch in the AP in epicardial cells. On a cellular basis the notch is caused by a transmural voltage gradient, originating from the presence of a notch in phase 1 of the AP (between maximum deflection and plateau-phase) of epicardial cells [11,12].

Besides experimental investigations, however, there is a lack of suitable computer models to study the effects of hypothermia on the ventricular myocardium in silico. Therefore, our main objective in this work was to develop a temperature-dependent model of a heterogeneous ventricular block to investigate hypothermia-induced electrophysiological effects from the cellular to the tissue level. To reach this goal a simplified biological, but computationally efficient minimal ventricular model (MV) for human ventricular APs, introduced by Bueno-Orovio et al.[13] was extended by temperature-dependent terms utilizing Q_10_ temperature coefficients. This extension allows investigating cooling induced effects on APs for epi-, M-, and endocardial cells. Based on this extended MV, a three-dimensional ventricular block model was developed to estimate temperature-induced effects on coupled myocardial cells in the myocardium. In order to accurately map the heterogeneous structure of the ventricle, three layers of distinct cell types (epi-, M-, and endocardial cell layer), different fiber orientation and the heterogeneous longitudinal, transversal and transmural conductivity were implemented. Additionally, temperature dependencies in conductivity and the transmural repolarization sequence were considered in the model. The simulation results are in good accordance with our experimental data obtained from cultures of chicken cardiomyocytes and to experimental data from literature.

Through the use of the developed model we are able to simulate and verify the impact of hypothermia on a three dimensional ventricular block. This impact is reconstructed from the bottom up: from electrophysiological changes on the cellular level to the resulting effects on the ECG. This model therefore offers a new opportunity to investigate electrophysiological changes in silico in the ventricular myocardium and their impact on ECG parameters under hypothermic conditions as they occur, for example, during TTM or accidental hypothermia.

## Material and methods

To reproduce the process of developing a temperature-dependent ventricular myocardial tissue model, our method comprises two main sections: The first section describes the experimental setup of cell cultivation and a feature extraction modality to determine the temperature coefficients Q_10_, which are needed to estimate cooling-induced alterations of intrinsic AP parameters of myocardial cells. These temperature coefficients are also essential for the second, the modeling section. This part includes the development of a general model and the functional design of a 3D ventricular block model to simulate temperature dependent alterations of the AP between 27°C and 37°C.

### Experimental setup

As described in [14], surface field potentials (FPs) recorded from n = 18 primary cultures of chicken cardiomyocytes at temperatures in the temperature range from 27°C to 37°C were analyzed. For extracellular recordings we used planar microelectrode arrays (MEAs), and a suitable recording system (Multi Channel Systems, Reutlingen, Germany). The detailed process of cell cultivation and signal registration is shown in S1 Text.

#### Estimation of temperature coefficients

A common measure to analyze and compare temperature-induced changes of physiological processes in a biological system is the temperature coefficient Q_10_ [15] which indicates the effect of decreasing or increasing the temperature by 10°C on the observed process and is defined as

$$Q_{10}={\left(\frac{X_{R}}{X_{A}}\right)}^{\left(\frac{10}{T_{R}-T_{A}}\right)}$$ (1)

where X_R_ denotes the value of the process at reference temperature T_R_ and X_A_ the value at the actual prevailing temperature T_A_. In our model, Q_10_ values are used to estimate cooling-induced alterations of intrinsic AP parameters of single myocardial cells. Due to a linear correlation between intrinsic features of APs and FPs [16] Q_10_ values of APs can be estimated from FPs registered from cardiac cell cultures at different temperature levels. In our study we applied a three-step procedure to calculate the temperature coefficients Q_10_:

1. Calculating relative FP parameter changes between two temperature steps for each single electrode of a MEA for each cellular preparation separately.
2. Estimating the median relative FP parameter change with reference to a temperature of 37°C for each cell layer including all electrodes of the MEA (see S1 Fig).
3. Using the results obtained from step 1 and 2 for all n = 18 primary cultures of chicken cardiomyocytes and applying non-linear least squares analysis for curve-fitting to compute Q_10_ values according to the equation:
  $$X_{A}=Q_{10}^{(T_{A}-T_{R})/10}$$ (2)

where X_A_(T) defines the observed relative temperature change based on the physiological reference tissue temperature T_R_ of 37°C and T denotes the investigated temperature. The formula is obtained by rearranging Eq (1) where X_R_ was set to 1 (relative reference point at 37°C).

According to this calculation procedure we were able to obtain Q_10_ values in a range of 37°C down to 23°C for the intrinsic FP parameters FP_rise_, FP_dur_ and FP_min_ of 0.61, 0.61 and 1.2 (RMSE: 0.12, 0.1 and 0.07), respectively, where FP_rise_ correlates linearly to the AP rise time (AP_rise_), FP_dur_ to the APD and the height of negative peak of the FP (FP_min_) presumably to the maximum upstroke velocity of the AP (V_max_) [16]. Additionally, Q_10_ values for the beating rate (Q_10_ = 0.28, RMSE = 0.7) and CV (Q_10_ = 1.5, RMSE = 0.04) of the studied cell layers were calculated. A CV value was estimated based on the local gradient of the wavefront arrival time detected at the single electrodes of the MEA as described in [14]. All obtained Q_10_ values show strong accordance with data from literature (see S2 Text).

### Design of the 3D ventricular block model

**Fig 1:**
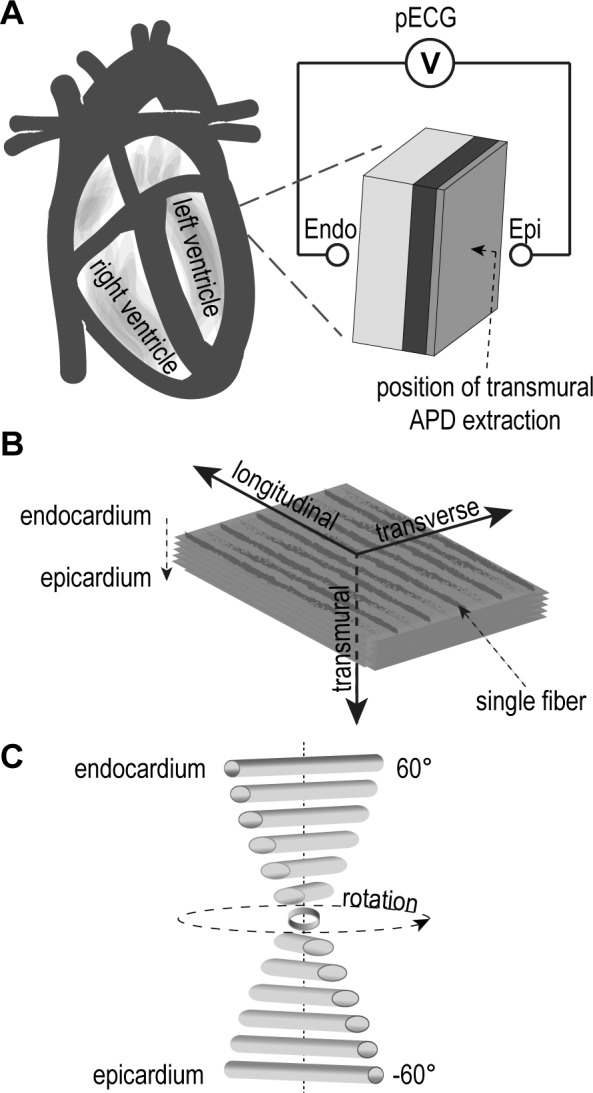
Schematic illustration of simulation setup. (A) the extracted virtual ventricular wedge and the pECG setup, (B) the orientation in longitudinal (fiber direction of the sheet), transverse (parallel to fibers of the sheet) and transmural direction (from endocardium to epicardium) and (C) helical transmural fiber orientation.

For simulation of hypothermia-induced effects in cardiac tissue, in particular for the formation of the J-wave, a myocardial tissue block of 2x2cm length/width with 10mm wall thickness was chosen. This tissue block corresponds well to a human myocardium selected approximately 1.5 cm below the mitral hinge line of the left ventricle of an adult heart [17] being in good accordance with data shown in [18]. A schematic illustration of the tissue block is depicted in Fig 1A. The ventricular wall of the human heart was modeled as a three-layer compartment structure composed of 10% epicardial cells, 30% M cells and 60% endocardial cells as described by Drouin et al. [19] and anisotropic conductivity with respect to the fiber orientation (longitudinal, transversal and transmural). This orientation is shown in Fig 1B. Additionally, the transmural fiber orientation was considered as a helical structure where the fiber angles ranged from -60° to +60° from epicardium to endocardium to achieve a closer approximation of the model to real tissue conditions (Fig 1C). This specific orientation is described for the left ventricle in [20–22]. The monodomain equation was used for modeling the electrical excitation to determine the electrical propagation through the virtual ventricular tissue block (see S1 Text). Analogous to [23], our cellular model is based on the MV model for ventricular action potential in human tissue [13]. This model enables simulations on tissue level for the human ventricle considering the three (epicardial, endocardial and midmyocardial) cell layers. To simulate the formation of hypothermia-induced J waves, it is important to emulate the electrophysiological conditions in the left ventricular as accurately as possible. This implies additional necessary adjustments in the 3D tissue model such as the distinct intramural CVs in x, y and z direction and an adaption of the transmural repolarization time of the three cell layer compartments.

#### Approximation of distinct intramural CVs

It is a well-known fact that anisotropy in the myocardial tissue results in distinct intramural activation times. This is manifested in different CVs between fiber direction (longitudinal), parallel to fibers (transverse) and from endocardium to epicardium (transmural). In more detail, there does exist a decrease in transverse direction compared to longitudinal direction by a factor of two and a reduction in transmural compared to CV in fiber direction by a factor of approximately four [24,25]. Additional heterogeneity in CV is evident between M and the epicardial cell layer based on a reduction in expression of the gap junction connexin gene Cx43 [8,26–28]. A common approach to accomplish experimentally observed CVs is to vary D [29]. As a consequence, distinct CVs were considered in the model by adapting the diffusion coefficients of the different cell types and different space directions. Table 1 summarizes the different CVs in the ventricular wall for distinct cell types and excitation directions based on literature and the calculated diffusion coefficients D at a prevailing temperature of 37°C (*D*_37_). However, CV values for transmural epicardial excitation were not available in literature. Therefore, we approximated the epicardial transversal velocity by the half of the epicardial longitudinal velocity analogous to the transverse CV of endocardial and M cells. Note that the transmural excitation has a known velocity of approximately 17cm/s [30,31]. However, Poelzing et al. [27] reported a reduction of CV between the M region and the epicardial cell layer at about 25% which is attributable to an intercellular uncoupling in the subepicardial layer. Therefore, we adapted the transmural CV of the endocardial and the M region (17.3 cm/s) and the transmural CV of the epicardial layer (13.8 cm/s) to accomplish this distinct transmural CV. However, by increasing the CV in epicardium and M layer and by decreasing velocity in epicardium a global transmural velocity (endo-epi) of 17 cm/s was still maintained. Finally, all coefficients for each cell type and fiber direction were determined, utilizing a one-dimensional strand model with a length of 20mm of the respective cell type with dt = 0.01ms and ds = 0.2mm. CV was determined between 4mm and 16mm relative to the start of the strand. The virtual strand was paced with a cycle length (CL) of 1000ms until the steady state was reached.

**Table 1:** Heterogeneous conduction velocities (CVs) for different ventricular cell layers and directions and the corresponding calculated diffusion coefficients at 37°C estimated for 3D simulations of a ventricular cell block.

Cell type	Fiber direction	CV [cm/s]	Diffusion coefficient D_37_ [cm^2^/s]
Endocardial M	longitudinal	67 [30]	0.9 (Endo)
			0.764 (M)
	transversal	30 [24], [25]	0.255 (Endo)
			0.236 (M)
	transmural	17.3 [30,31]	0.124(Endo)
			0.124 (M)
Epicardial	longitudinal	54 [32]	0.692
	transversal	25	0.214
	transmural	13.8	0.102

#### Adjustment of transmural repolarization time

APD in isolated epicardial, M or endocardial cell studies are observed to have significantly different durations [33,34]. In the MV model, the APD of epicardial and endocardial cells show similar duration where M cells demonstrate a longer APD (see model of Bueno-Orovio et al. for human ventricular cells [13]). However, normal intercellular electrically-coupled cells tend to average distinct myocardial action potentials, resulting in less significant APD gradients between epicardial, M and endocardial cell layers [35,36]. In left ventricular tissue the APD of the M region shows the longest durations, followed by the APD of the endocardium. The epicardium demonstrates the shortest duration. A gradual decrease of the APD exists between M and the epicardial cell layer as well as M and the endocardial layer respectively based on the aforementioned averaging effects of the APDs [8,27,28]. According to the literature, the approximated ratio between (i) maximum APD in M cells and minimum APD in endocardial cells, and the (ii) maximum APD in M cells and the epicardial cell layer is about 1.04 and 1.3, respectively [8,27,28,36]. In our model this averaging effect between distinct cell layers was also present and led to incorrect APD ratios between the M-subendocardial and M-subepicardial layers where the epicardium shows longer APDs than the endocardium. As a consequence, we adjusted the initial MV model parameters to preserve physiological APD ratios. Since APD is primarily controlled by the time constants t_w+_, t_si_, and t_so_ in the MV model [13] we introduced the factor cAPD for endocardial and epicardial cells to maintain the above approximated physiological APD ratios of about 1.04 and 1.3, respectively. Eq 4 demonstrates the APD-ratio-adjustment.

$$\begin{array}{l} C_{j}↔(\tau_{w+},\tau_{si},\tau_{so})\cdot \mathrm{cAP}D_{j}\\ j=epicardial,endocardial\\ cAPD_{j}=\{\begin{array}{ll} 1.05 & j=epicardial\\ 1.40 & j=endocardial \end{array} \end{array}$$ (3)

This modification results in a physiologically reasonable APD ratio between distinct cell layers for simulations of heterogeneous 3D left ventricular tissue. An illustration of the APD between distinct cell layers before and after modification is shown in S3 Fig.

### Temperature dependent modifications

As introduced, we modified the MV model to simulate hypothermally-induced intrinsic variations of the AP morphology with the objective to study cardiac tissue under hypothermic conditions. More specifically, hypothermia induces a prolongation of the APD [37,38], a prolongation of the AP rise time, a decrease of V_max_ [37,39,40] and an increase in the amplitude of the epicardial notch [12]. These variations in AP morphology are mainly caused by temperature-dependent changes in ion channel kinetics. Therefore, we adapted the ion channel kinetic of the three virtual currents of the MV model by modifying multiple time constants *τ*_*x*_ by fitting the estimated Q_10_ values. The underlying basic equation for modifying time constants *τ*_*x_modified*_ of the MV model is given as

$$\tau_{x\_modified}=\frac{\tau_{x}}{Q_{10_{x}}^{(T_{R}-T)/10}}$$ (4)

where *τ*_*x*_ describes the original time constant of the MV, Q_10_ is the calculated temperature coefficient for the respective feature being modified. T is the prevailing hypothermic temperature and T_R_ is the reference temperature which equals 37°C.

Analogously to the determination of the parameter D (see section “Approximation of distinct intramural CVs”), $Q_{10_{x}}$ values were calculated utilizing a one-dimensional strand model (2cm length, dt = 0.1ms, ds = 0.2mm, CL = 1000ms) until a steady state was reached. *Q*_10*x*_ values for the required time constants *τ*_*x*_ to modify APD, V_max_ and epicardial notch as well as $Q_{10_{D\_celltype}}$ for diffusion coefficient D to modify CV for distinct cell types were, respectively, adapted to fit the Q_10_ values determined from our *in vitro* experiments (prolonged AP_dur_, decreased CV) and literature data (increased AP notch, decrease in V_max_) identical for each cell layer (epicardium, M layer, endocardium). The calculated Q_10_ values are summarized in Table 2.

**Table 2:** Calculated Q_10_ temperature coefficients for APD, V_max_ time, AP notch and CV for epicardial, M and endocardial cells respectively.

	APD			V_max_		AP notch	CV
	τ_si_	τ_so_	τ_wp_	τ_fi_	τ_vp_	τ_s_	D
Epicardial	0.69	0.69	0.69	0.57	0.57	0.24	1.35
M	0.53	0.53	0.53	0.67	0.67	0.53	1.39
Endocardial	0.58	0.58	0.58	0.60	0.60	0.58	1.40

#### APD prolongation

As described by the work of Bueno-Orovio et al., APD is primarily controlled by the time constants *τ*_*w*+_, *τ*_*si*_ and *τ*_*so*_ [13]. A modification of these three parameters as denoted in Eq 4 results in an APD prolongation with falling temperatures. The temperature coefficients $Q_{10\tau_{w+}}$, $Q_{10\tau_{si}}$ and $Q_{10\tau_{so}}$ were adapted to obtain a Q_10_ value for APD of 0.61, referring to the Q_10_ kinetics of the FP parameter FP_dur_ (see Table 2) which correlates to the APD [16]. In the literature, however, we found Q_10_ values of APD in the range of 0.44 to 0.83 (see S2 Text).

#### Prolongation of AP_rise_ time and decrease of V_max_

A modification of *τ*_*fi*_ and *τ*_*v*+_ with the Q_10_ kinetics for V_max_ enhances the AP_rise_ time and lowers V_max_ with decreasing temperature. The temperature coefficients $Q_{10\tau_{fi}}$ and $Q_{10\tau_{v+}}$ were adapted to obtain a Q_10_ value for V_max_ of 1.68 (see Table 2). This temperature coefficient for V_max_ was evaluated based on literature data (see S2 Text)

#### Increase in the amplitude of the epicardial notch

As it is known, the spike-and-dome action potential morphology responds to falling temperatures with an increase in the notch amplitude and width. The response is more prominent in the epicardium compared to the endocardium. This hypothermia-induced cellular electrophysiological variation was proposed as basis for the J wave [12]. The coherence of epicardial notch morphology and J wave elevation was also demonstrated by Bueno-Orovio et al. [41]. Such cellular variations in the amplitude of the notch can be modeled by adapting the time constant *τ*_*s*_ of the used MV model with distinct Q_10_ kinetics for each cell type according to Eq 4. Based on plotted experimental data in the work by Yan and Antzelevitch [12], the transmural measured J-wave at 29°C has approximately twice the amplitude compared to 37°C. To reproduce this data in our 3D model, the following $Q_{10\tau_{s}}$ values were selected to modify *τ*_*s*_ for distinct cell types and resulted in a more prominent hypothermia-induced increase in the epicardial notch compared to endocardial and M cell spike-and-dome action potential morphology (see Table 2).

#### Reduction of CV

The resistance of intercellular cardiac gap junctions (R_gj_) is highly temperature dependent and is manifested in an inverse correlation between junctional resistance and temperature [42,43]. As discussed and due to the fact that the intercellular resistance is an important factor for CV, an increase of the R_gj_ leads to a reduction of the CV. Based on the definition of D (see S1 Text), a hypothermia-induced reduction of CV is modeled by modifying the diffusion coefficient at 37°C (D_37_) with the Q_10_ kinetics as follows:

$$D_{celltype\_modified}(T)=\frac{D_{37\_celltype}}{Q_{10_{D\_celltype}}^{(T_{R}-T)/10}}$$ (5)

Analogous to time constants *τ*_*x*_, $Q_{10_{D\_celltype}}$ is the calculated temperature coefficient for D for a cell type being modified, T is the prevailing hypothermic temperature and T_R_ is the reference temperature which equals 37°C. This modification was performed for each cell type (see Table 2) to obtain a Q_10_ value of 1.5. This value was experimentally determined for the temperature coefficient for CV and is in good accordance with the literature (see S2 Text).

#### Adaption of the cycle length (CL)

The CL corresponds to the RR interval and describes the time between two heart beats. CL was fixed to 1000ms within our simulations. However, the CL depicts an inverse correlation with the temperature [44]. Based on our in-vitro experiments we determined a Q_10_ value of 0.28, which is in good accordance with the literature (see S2 Text). Additional simulations employing the ventricular block model were performed with a temperature dependent CL in order to observe the impact of hypothermia-induced increase in CL compared to a fixed CL. The CL for each temperature level was adapted according to the following equation:

$$CL(T)=\frac{1000ms}{0.28^{(T_{R}-T)/10}}$$ (6)

T is the prevailing hypothermic temperature and T_R_ is the reference temperature which equals 37°C.

### Validation

For validation of the model, the simulated results were compared to literature at cellular and tissue level. Variations in AP parameters and in the ECG of the ventricular block were reviewed and interpreted on cellular and tissue level, respectively.

#### Determination of AP parameters

The APD was defined as the interval between 10% AP onset and 90% of repolarization (APD_90_). The following parameters were used to describe changes in the epicardial notch, responsible for the genesis of the J wave: the AP notch magnitude, the duration of the notch as phase 0 to phase 2 interval (time between the first two peaks of the derivative of the AP) and the notch index which approximates the area of the notch (notch magnitude × notch duration) [11]. Transmural dispersion of repolarization (DOR) was defined as the time between longest and shortest transmural APD. V_max_ refers to the maximum upstroke velocity (*dv*/*dt*)_*max*_ of the AP-slope of phase 0 (rapid depolarization phase). A graphical illustration of a single AP complex and the determined parameters is shown in S2 Fig.

#### Pseudo ECG (pECG) calculations

In this study, we estimated pECGs based on the simulation results of the virtual ventricular block under normal and hypothermal temperatures. This allows for a comparison of our simulation to those in literature. For simulation of the pECGs the similar lead field concept as described in [45,46] was utilized (see S1 Text). The transmural bipolar pECGs were calculated from the center of the ventricular block where the virtual electrodes were placed at a distance of 10mm from the epicardial and endocardial surface (Fig 1A). The virtual sampling rate was set to 1 kHz.

#### Determination of pECG parameters

The comparison of results from our 3D model with literature data requires the determination of specific features of the computed pECG. To investigate the J wave shape, the amplitude was measured between baseline and maximal deflection of the J wave. To determine the hypothermia-induced alteration on the morphology of the repolarization wave (T-wave), the T-peak to T-end interval (TpTe) was determined by the distance of the peak of the T-wave to the end of the T-wave as described in [47]. The interventricular excitation time (QT interval) was defined by the time between the onset of the Q wave and the point of 90% recurrence of the T wave. The QRS-interval was determined by the time difference between the maximal deflection of the simulated Q and S wave. A graphical representation of a single pECG complex and the determined ECG parameters is shown in S2 Fig.

#### Numerical methods

Partial differential equations were numerically solved using the forward Euler-method with uniform spatial and temporal resolution of 0.2mm and 0.01ms, respectively. The initial conditions of the extended MV model for epicardial, endocardial and midmyocardial cells were set identical as specified in the original work of Bueno-Orovio et al. [13] for human ventricular tissue. Finite differences formulations for inhomogeneous anisotropic bioelectric media proposed by Saleheen [48] were employed to solve 2D and 3D problems with anisotropic conductivities. Non-flux boundary conditions (isolating Neumann boundary) were set for all simulations in 1D, 2D and 3D.

## Results

**Fig 2:**
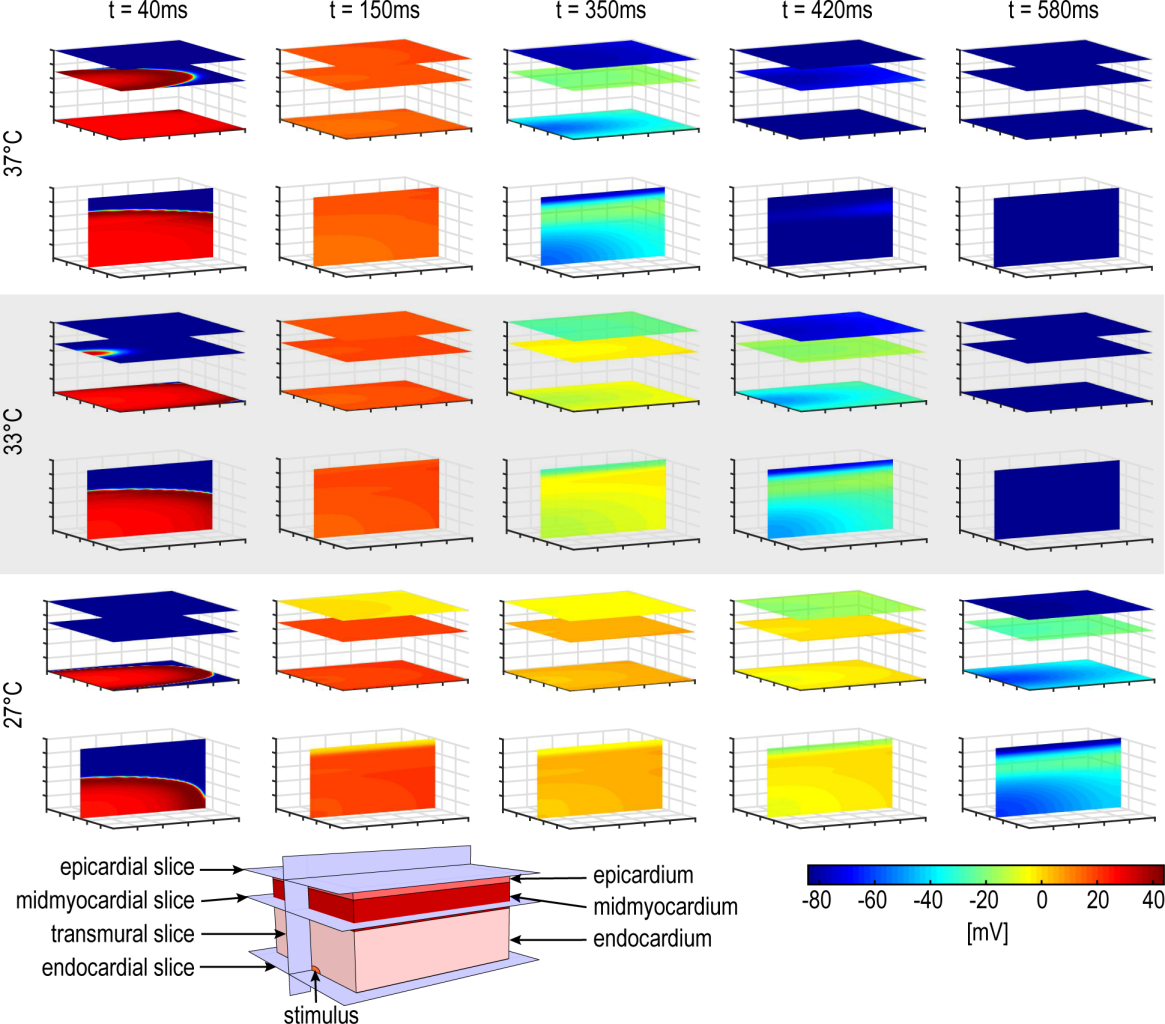
Visualization of excitation propagation of the simulated myocardial block for selected timestamps and temperatures. The colors illustrate the cellular membrane potential, where dark blue represents repolarized cells and red the maximum depolarized cells (phase 1 of the AP). The dimension of the simulated myocardial block is 2x2x1cm where the thickness of the epicardial, midmyocardial and endocardial layers are 1mm, 3mm and 6mm, respectively. The stimulus was set in the middle of the left edge of the endocardium. The pictures include the visualized propagation of the outermost endocardial and epicardial layer respectively, the midmyocardial layer with the longest APDs (upper view each) and the transmural excitation in the middle of the block (bottom view each). At time point t = 40ms the hypothermal-induced slowed excitation propagation in all directions is evident. At t = 150ms the myocardial cells are in the plateau phase under normal and hypothermal conditions. A cooling- induced APD prolongation can be observed for the timestamps t = 350ms, t = 420ms and t = 580s where the epicardial layer reveals the shortest repolarization time followed by the endocardial and midmyocardial layer.

Using the modified MV-model at cellular and tissue level, a ventricular block was simulated and prominent electrophysiological parameters such as the APD, QT-interval or J-wave were computed. The simulated block was virtually cooled down in steps of 2°C starting at 37°C until 27°C was reached, and the transmural pECG was calculated for each temperature level. Fig 2 illustrates a single AP cycle (excitation, depolarization, plateau phase, repolarization) of myocardial cells in a simulated ventricular tissue block. Cooling-induced effects of decelerated excitation and prolonged APD are obvious. Additionally, varying APD for the three distinct cell types (epicardial-, midmyocardial-, and endocardial layer) could be identified where the epicardial layer shows the shortest, and the midmyocardial layer the longest, activation period between excitation and repolarization phase.

### Effects of temperature on APD and QT-interval

**Fig 3:**
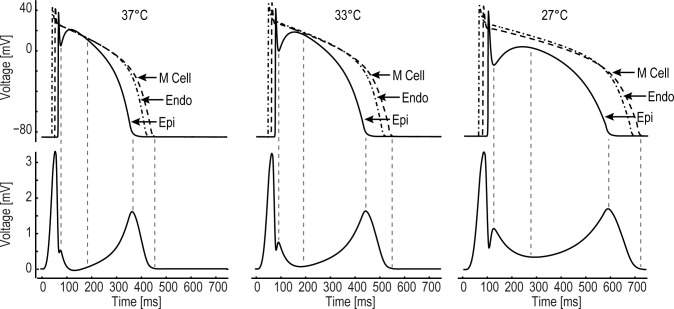
Correlation of the voltage gradients between the myocardial layers and the T wave morphology. Top: Transmembrane action potentials (AP) representing the outermost epicardial and endocardial layers and the midmyocardial layer with the longest APD for different temperatures of 37°C, 33°C and 27°C. Bottom: calculated pECG across the simulated block. Additionally, a coincident time behavior of the epicardial notch and electrocardiographic J wave as well as repolarization of distinct cell layers and T wave are clearly evident.

Under physiological conditions the morphology and time behavior of the T wave of transmural recorded pECGs is attributable to distinct morphologies and time characteristics in phase 2 (plateau) and phase 3 (repolarization) of epi-, M- and endocardial cells. In particular, the T wave starts with the AP plateau phase separation of the epicardium from the M region, reaches its peak at the time of full repolarization of the epicardial cells and ends with the repolarization of the M cell layer [49]. As aforementioned, hypothermia induces a prolongation of the APD of epi-, M- and endocardial cells. As a consequence, hypothermia prolongs the QT interval. Fig 3 shows the simulated changes in AP morphology at selected temperature levels and calculated transmural pECG for fixed CL. The cooling-induced APD prolongation and the associated increase of the QT interval and a coincident time behavior of repolarization of distinct cell layers and T wave are illustrated.

**Fig 4:**
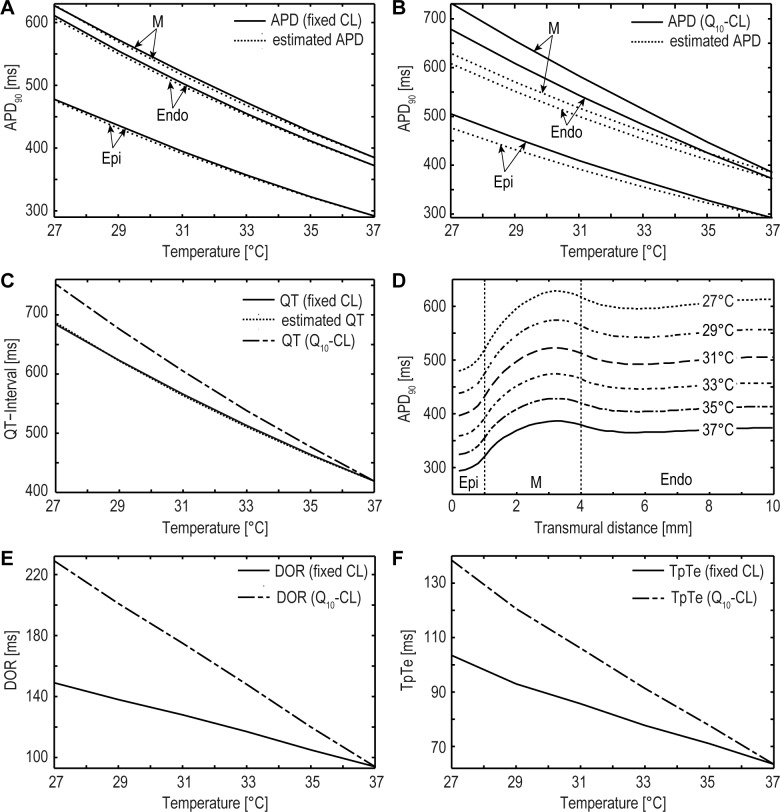
Effects of temperature on APD, QT interval, DOR and TpTe. (A-B) Temperature dependent prolongation of the APD90 compared to the calculated Q_10_ curves (Q_10_ = 0.61) of the epicardial, the midmyocardial and the endocardial layer. A hypothermia-induced prolongation of APD using the model is evident, however, this prolongation is more prominent for temperature dependent CL (Q_10_-CL) than for fixed CL. (C) Prolongation of the QT interval induced by falling temperatures (calculated based on the simulated pECG for fixed CL and temperature dependent Q_10_-CL, respectively). (D) Visualization of the transmural averaging effect of APD between coupled cells in the simulated ventricular wedge. All APDs were selected from the center of the epicardial surface to the center of the endocardial surface along the same z-axis until a steady state was reached. The transmural heterogeneity was modeled by a sub-layer of epicardial cells (10% of the total thickness of the ventricular wall, between 0 to 1mm), midmyocardial cells (30%, between 1 to 4mm) and endocardial cells (60%, between 4 to 10mm). (E-F) Indices for transmural dispersion calculated from simulated APD values (DOR) and pECG data (TpTe), showing a linear correlation with decreasing temperature levels. As expected from the simulated results for transmural heterogeneous APD, the variations of APD are higher for temperature dependent Q_10_-CL than for fixed CL.

Fig 4A and 4B illustrates the simulated temperature-induced prolongation of the APD for each myocardial region (epi-, M-, endocardial) in comparison to the rate of change for the experimentally determined temperature coefficient (Q_10_ = 0.61). This comparison was computed for fixed and temperature-dependent CL (Q_10_-CL), starting with the calculated APD at 37°C each. It is obvious that the simulation data almost corresponds to the experimentally determined temperature coefficient for fixed CL where the APD is the shortest in the epicardial region followed by that of the endocardium. The M region yields the longest APD (Fig 4A). For Q_10_-CL the deviations from the estimated values are more prominent for M and endocardial cells than for the epicardial region (Fig 4B). An impact of CL on APD can also be found in the literature [19,33,50]. APs were selected from the middle of the simulated block of ventricular tissue (Fig 1A) for the outermost epicardial layer, the M region with the longest obtained APD and the outermost endocardium. As previously discussed a prolongation of APD of myocardial cells induces an elongated QT interval. Fig 4C shows the QT interval based on the calculated pECG. Weitz et al. [51] reported an elongation of 135% for the QT interval during hypothermia (400ms at 37°C to 540ms at 33°C). Similar results are also described by Khan et al. [52]. Those findings are comparable to our simulation results at 33°C (fixed CL: 419ms at 37°C to 513ms at 33°C, 123% increase; Q_10_-CL: 419ms at 37°C to 538ms at 33°C, 129% increase). For severe hypothermia our simulations reveal a QT interval of 685ms (fixed CL) and 752ms (Q_10_-CL). This represents an increase of about 170 percent (fixed CL: 163%, Q_10_-CL: 179%) compared to the QT interval at 37°C. Similar to the APD, QT prolongation also matches more precisely the experimentally determined rate of change of the temperature coefficient Q_10_ = 0.61 for fixed CL than for Q_10_-CL. The aforementioned transmural averaging effect of APD between coupled cells in tissue at different temperatures is shown in Fig 4D. APDs in the epicardial as well as the endocardial layer successively increase until the peak value is reached in the M-region. Indicators often used to describe this transmural dispersion of repolarization are DOR (based on APD values) and TpTe (based on ECG data). In our model DOR increases from 94ms to 149ms during cooling (from 37°C down to 27°C) for fixed CL and from 94ms to 229ms for temperature dependent CL, respectively (Fig 4E). In the observed temperature range TpTe increases from 63.5ms to 103.5ms for fixed CL and from 63.5ms to 138.4ms for temperature dependent CL, respectively (Fig 4F).

### Effects of temperature on epicardial AP notch and J-wave

**Fig 5:**
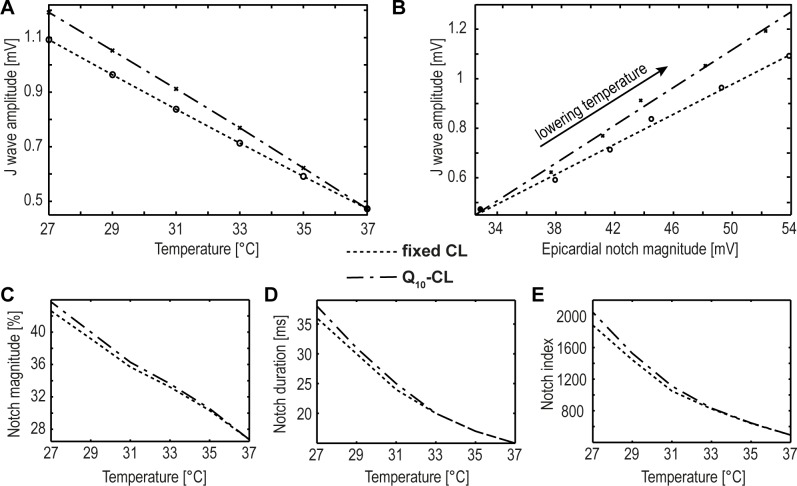
Effects of temperature on epicardial AP notch and J-wave. (A) Correlation between J wave amplitude and temperature. (B) Correlation between J wave amplitude and amplitude of the epicardial notch when decreasing the temperature from 37° down to 27°C. The dotted lines indicate the linear regression curves (r = -0.99 (A), r = 0.99 (B)). In both experiments the J wave amplitudes were determined based on the simulated transmural pECG for both the fixed CL and temperature dependent Q_10_-CL. Notch magnitude as percentage of phase 0 amplitude (phase 1 magnitude/phase 0 amplitude ×100) (C), notch duration (D) and notch index (E) increase significantly during cooling.

The J-wave is characteristically seen in ECG recordings under hypothermal conditions where the amplitude of the J-wave inversely correlates to temperature. Based on the work of Yan et al. [12] the ratio between J wave amplitudes at 29°C versus 37°C is approximately two. Our simulated pECGs signals show a ratio of 1.92 for fixed CL and 2.07 for temperature dependent CL, respectively. Our data thus confirms this correlation, showing a linear relationship between decreasing temperature and increasing amplitude of the J-wave (Fig 5A). As introduced, the J-wave originates from a cooling-induced increase in the notch of the epicardial AP. Additionally, coinciding time characteristics of the epicardial notch and electrocardiographic J-wave can be observed (see Fig 3). Analogous to the experimental setting of Yan et al. [12] our simulated data reveals a highly significant correlation (r = .99, p<0.01) between epicardial action potential notch and J wave amplitudes during the cooling process (see Fig 5B). Analogous to experimental data published by Gurabi et al. [11] our data also reveals a significant increase in the notch index, the notch magnitude and the notch duration in epicardial cells (Fig 5C–5E).

### Effects of temperature on QRS interval

**Fig 6:**
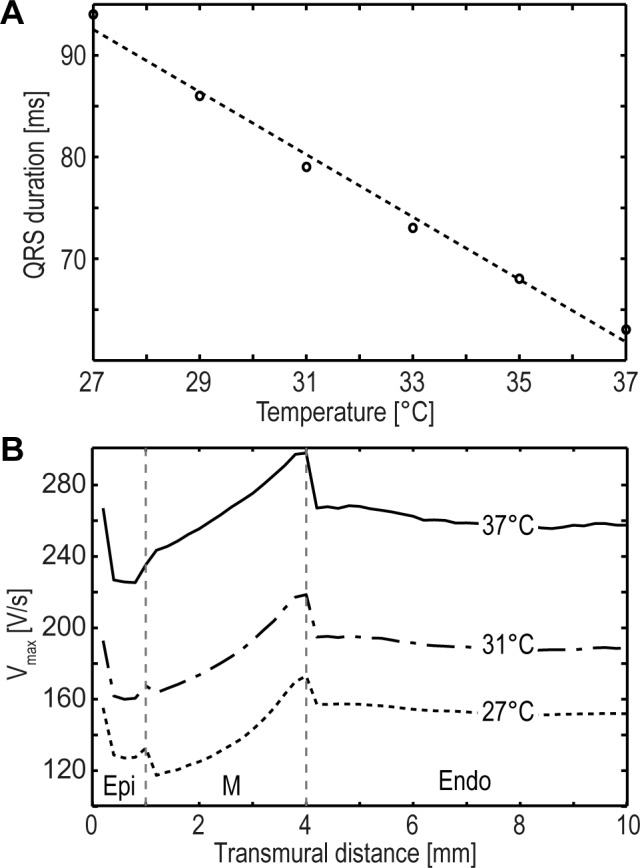
Effects of temperature on QRS interval and V_max_. (A) Correlation between QRS duration and temperature. A cooling-induced prolongation of the QRS interval is evident. The dotted line represents the linear regression curve (r = -0.99, p<0.01). (B) Transmural distribution of V_max_ for different temperatures (37°C, 31°C, 27°C). The cooling-induced decrease of V_max_ for all cellular regions is obvious. Additionally, the heterogeneity of V_max_ based on divergent cell types is shown where the M region yields the highest V_max_ value followed by the endocardium and the epicardium.

Hypothermia induces a prolongation of any detectable electrocardiographic intervals including the QRS interval. Our model confirms this known relationship between QRS duration and temperature. A prolongation of 3.07ms/°C was determined (Fig 6A), corresponding to a 4.9% change with respect to the baseline value at 37°C. In comparison, Kågström et al. [53] also observed an increase in QRS duration for guinea pigs of 0.8±0.3 ms/C°, corresponding to a 2.8% change w.r.t. the baseline value at 38°C. In the model no significant difference between fixed CL (CL = 1000ms) and temperature-adjusted CL was seen.

### Effects of temperature on V_max_ and AP_rise_ time

The prolonged AP_rise_ time and a decrease of V_max_ are effects of hypothermic myocardial tissue. However, these two morphologic AP characteristics deviate between different myocardial cell types. Fig 6B shows the transmural changes in V_max_ for different temperatures (37°C, 31°C, 27°C). The cooling induced decrease of V_max_ [37,39,40] is obvious. In addition, the cell type based heterogeneity of V_max_ is visible, where V_max_ of the M region shows the highest value, followed by the V_max_ of the endocardium. The epicardium demonstrates the lowest V_max_. This pattern of V_max_ heterogeneity within distinct cell types is comparable to experimental studies in the human ventricle [19,54]. In addition to the aforementioned transmural averaging effect of APD in normal intercellular electrically-coupled cells, a transmural averaging effect of V_max_ is also present in our model. At the boundaries between distinct cell types (endocardium-midmyocardium, midmyocardium-epicardium) and between epicardium and isolating border zone, a jump in V_max_ is observed. This can be explained by the sudden change in D and the associated conduction change, and is seen in the boundary-adjacent simulated slice (single slice ds = 0.2mm). In our model, the calculated Q_10_ value (median) is 0.62 for AP_rise_ and 1.7 for V_max_, respectively. In comparison, our experimentally determined Q_10_ value for FP_rise_ is 0.62 and for FP_min_ 1.2, where FP_rise_ correlates with AP_rise_ and FP_min_ presumably with V_max_ [16]. In the literature, however, we found Q_10_ values of V_max_ in the range of 1.4 to 2.32 (see S2 Text).

## Discussion

### Myocardial cell model

The modified MV-model was introduced for simulating cooling-induced effects on the AP morphology at the cellular level. This model shows a good trade-off between a precise imitation of the cellular nature of cardiac tissue and demands on computational modeling and simulation and an adaption of this phenomenological human AP model to study electrophysiological mechanism of the human heart can also be found in the literature [41,55]. It also takes into account a precise physiological and anatomical description of epi-, M- and endocardial cells and tissue structure which is essential for building an extended ventricular simulation model. Cooling-induced alterations in AP morphology and CV are primarily attributed to modifications in cellular ion-channel kinetics. In literature a well-established concept used to describe temperature dependent alterations in cardiac models is the introduction of the so-called Q_10_ factors. In this approach, the mathematical description of the three virtual ionic currents (fast inward, slow inward and outward) of the selected and adopted MV model was extended according to the Q_10_ factors to simulate cellular channel kinetics in hypothermia. In comparison to [23], we solely changed multiple time constants of the four main variables of the original Bueno-Orovio model instead of the variables themselves. Note that the time constants are clearly associated with the activation or inactivation process of the gating variables. Using this approach (instead of adopting the model variables and ionic density currents as described by Fenton et al [23]) the absolute amplitudes of the AP do not decrease during temperature decrease. It is important to note that no significant decrease in the action potential amplitude in the range of 37° to 27°C has been reported in literature [39,40,56,57]. A further modification is required to reproduce the hypothermia-induced J-wave. As already discussed in this work, the J wave originates from a more prominent temperature-dependent variation in the notch of epicardial cells compared to endocardial and epicardial cells, respectively. This distinct variation is modeled by adjusting the time constant *τ*_*s*_ accordingly, which is necessary to induce the J wave in simulations of a 3D anisotropic ventricular block.

### 3D ventricular model

For a sufficiently precise simulation of electrophysiological mechanisms of the ventricular myocardium during cooling it is important to consider the biological structure, including different cell types and distinct fiber orientations and their heterogeneously intercellular conductivity in longitudinal, transversal and transmural fiber directions. The heterogeneities in conductivity, primarily based on distinct gap junctional properties, are represented by different CVs described by nine distinct D values. Intercellularly connected myocardial cells, however, implicate transmural averaging effects of APs. This effect leads to an incorrect sequence of APD of the different myocardial layers (epicardial, M, endocardial) in the simulations. By introducing the cAPD correction factor we are able to correct the APD of the different cell layers and maintain the physiological repolarization sequence (epi->endo->M). For the temperature dependent model description of an anisotropic 3D ventricular block it is essential to consider intercellular, cooling-induced effects, mainly characterized by a decrease in the gap junction conduction, and therefore leading also to a modulation of the CV. The values of the diffusion coefficient D were adjusted by the Q_10_ factors for simulating this cooling-induced decrease in CV. The approach of utilizing distinct temperature-dependent diffusion coefficients results in temperature-modulated alterations in CV without the necessity to modify the described temperature-dependent cellular model. The final model benefits from the ability to accurately reproduce hypothermia-induced effects from the cellular level by describing the morphologic changes of the APs of electrically coupled cells at the tissue level which simulate changes in the ventricular pECG. Moreover, the model has the ability to incorporate individual temperature distributions in time and space. All investigated parameters (APD, epicardial notch, V_max_, AP_rise_ time, QT-interval, QRS duration, J wave morphology) are in excellent accordance to literature.

### Hypothermia induced effects

#### Prolonged APD and QT interval

**Fig 7:**
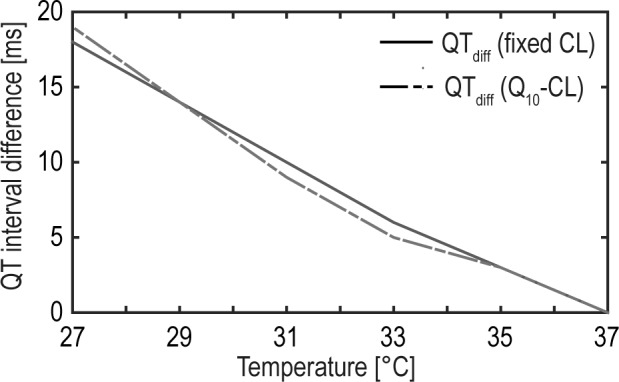
Impact of conduction slowing to the QT interval during hypothermia. The difference between considering and neglecting the cooling-induced decrease in conduction related to the hypothermia-induced QT interval prolongation is displayed. The underlying QT intervals were determined based on the simulated transmural pECG (fixed CL and temperature dependent Q10-CL).

Our simulation results have clearly demonstrated the expected hypothermia-induced prolongation of the APD at cellular level and the associated prolongation of the QT interval at tissue level. This prolongation effect, however, is more evident, taking into account a cooling-induced extension of CL compared to a fixed CL. The correlation between increasing CL and prolonged APD at hypothermic conditions coincides well with the observations of Bjørnstad et al. [40,58]. Inspecting the absolute values of prolongation of the QT interval in detail there was found a good correlation in the literature at mild hypothermia [51,52]. For severe hypothermia (<28°C) our simulations reveal a QT interval of approximately 700ms. Comparable data have been reported in the literature [59,60], although available data for severe hypothermia is limited. Nevertheless, an adaption of the QT interval during severe hypothermia can be easily achieved by an adjustment of the APD prolongation at reduced temperatures (temperature coefficients $Q_{10\tau_{w+}}$, $Q_{10\tau_{si}}$ and $Q_{10\tau_{so}}$). Transmural investigations of simulated APD showed inhomogeneous APDs depending on the myocardial cell type (epi-, M-, endocardial cells). Additionally, a transmural averaging effect of APD can be observed. This transmural APD dispersion is described by calculating DOR. Furthermore, transmural repolarization dispersion has a significant impact on the T-wave morphology. The parameter TpTe, therefore, may represent a distinct repolarization based on the ECG [61]. However, there is little evidence of DOR and TpTe during hypothermia in the literature. In turn, an inverse correlation to temperature may be expected as described in [47,61]. Nevertheless, our simulated data also confirms this inverse correlation of DOR and TpTe. Based on the functional separation of AP (modified MV model) and gap junction conductivity (adaption of D) the proposed model has the power to differentiate between the temperature influence on intracellular and intercellular level. This, for example, allows to investigate the effect of hypothermia-induced conduction slowing on the QT interval by setting the Q10 values modifying D to 1 (Eq 5). As a consequence, the temperature dependence of the gap junctions is switched off whereas the temperature sensitivity of the myocardial cells is still maintained. The result suggests that a temperature-induced decrease of conductivity prolongs the QT interval (Fig 7). Compared to the cooling-induced APD prolongation, however, the conductive slowing has a limited effect to the hypothermia-induced QT interval prolongation (maximum of approximately 20ms at 27°C).

#### Effects of temperature on epicardial AP notch and J-wave

The J-wave originates from a stronger cooling effect on the morphology (notch) of epicardial cells compared to M- and endocardial cells, and typically emerges in ECGs registered during hypothermia. Analogous to the experimental data of Yan et al. [12] and Gurabi et al. [11], our model demonstrates a temperature-dependent correlation of all investigated shape parameters of the J-wave (J-wave amplitude, AP notch magnitude, the notch duration, the notch index) in the simulated pECG.

#### Effect of temperature on QRS interval

As known, hypothermia induces a decrease in cellular conductivity. Such a decrease results in a slowed propagation of electrical conduction through the myocardium [9]. In the ECG slowed propagation is characterized by a QRS interval widening [10] and an inverse correlation of temperature and duration of the QRS interval was observed by Kågström et al. as well [53]. Our simulations confirmed this effect.

#### Effects of temperature on Vmax and APrise time

It is well known that V_max_ and AP_rise_ time correlates inversely with temperature [39,58]. The characteristics of these two parameters, however, are dependent on the myocardial cell types (epicardial, M, endocardial). Analogous to APD, V_max_ and AP_rise_ time are additionally influenced by transmural averaging effects of the electrically coupled cells. This results in transmural V_max_ heterogeneity. Our model has the capability to reproduce and confirm this transmural heterogeneity. In addition, our simulations suggest similar patterns for transmural V_max_ distribution during cooling (see Fig 6B). However, in literature there is little data available for V_max_ and AP_rise_ time as well as for the transmural dispersion of these two parameters during hypothermia.

### Limitations

In ECG, the morphology of the ST-segment and the T-wave are highly sensitive to the characteristics of the cellular AP phase 2 and 3 (plateau phase and repolarization phase) of distinct cell types. Our model computes the APD, primarily composed through the phase 2 and 3 duration values. The exact biological morphology of the plateau phase of epi-, M- and endocardial cells during cooling was neglected. Visual inspections of APs in literature, however, moderate cooling-induced variations in the plateau phase of distinct cell types [11,12]. In addition to the computation of the APD, exact descriptions of AP phase 2 and 3 are required to obtain a better agreement with experimental data. This primarily applies to hypothermia-induced ST-segment elevation or T-wave formation [6].

This work classified repolarization sequences progressing in the form epicardium–endocardium–M cells as physiological. This concept derives from isolated preparations, such as myocardial slices, wedge preparations or disaggregated myocytes [8,27,28,36]. In the intact heart this concept of differences in the transmural repolarization times is under debate [62]. The proposed model, however, has the ability to modify APD ratios by simply varying the factor cAPD.

### Conclusion

Hypothermia has a significant effect on the electrophysiology of the heart. Besides the previous experimental studies described in literature there are, however, a lack of computational studies describing the complex physiological process of hypothermally induced electrophysiological variations in ventricular tissue. In this work, we have proposed a new temperature sensitive 3D computer model describing hypothermal-induced electrophysiological alterations in ventricular myocardium considering a high level of detail (different cell types, heterogeneous conductivities, distinct fiber orientations and repolarization sequences). This includes alterations in AP notch, APD, AP_rise_ time, V_max_, DOR, J-wave, QRS interval, QT interval and TpTe. Additionally the proposed model allows a separate analysis between the temperature changes on intracellular level (action potential) and intercellular connections (gap junctions). The simulations are in excellent accordance to our experimental data and literature data. The introduced model can be used as a starting point for investigating electrophysiological mechanisms from cell to tissue level observed in cardiac tissue during TTM or accidental hypothermia. In particular, in clinical practice this data may help to further improve the therapeutic outcome or to better understand and handle possible undesirable effects of hypothermia, e.g. in arrhythmogenesis [63–66].

## Supporting information

S1 Text Supplemental methods. Additional Information about MEA Recordings, approximation of distinct intramural CVs, pseudo ECG calculations.
(PDF)

S2 Text Q_10_ values extracted from literature for APD, V_max_, CL and CV. (PDF)

S1 Fig Boxplots of the relative changes of FP parameters (FP_rise_,FP_dur_,FP_min_) and CV. (PDF)

S2 Fig Schematic illustration of a single AP complex and of a single pseudo ECG complex. (PDF)

S3 Fig Simulated transmural APs without APD correction. (PDF)

S1 Table Detailed information about all absolute values. Detailed information about all absolute FP values and CV estimated at different temperatures for n = 18 cell layer.
(PDF)

## Abbreviations

AP: action potential
AP_rise_: action potential rise time
APD: action potential duration
CL: cycle length
CV: conduction velocity
D: diffusion coefficient
DOR: transmural dispersion of repolarization
FP: field potential
FP_rise_: field potential rise time
FP_dur_: field potential duration
FP_min_: height of negative peak of the field potential
MEA: microelectrode array
MV: minimal ventricular model
pECG: pseudo electrocardiogram
Q_10_: temperature coefficient
TTM: targeted temperature management
TpTe: T-peak to T-end interval
V_max_: maximum upstroke velocity of the action potential
