# Supplementary material for: Modeling hypothermia induced effects for the heterogeneous ventricular tissue from cellular level to the impact on the ECG
Source: PLoS One. 2017 Aug 16;12(8):e0182979. doi: 10.1371/journal.pone.0182979 (PMC5558962; doi:10.1371/journal.pone.0182979)
Supplement: S1 Text — Additional Information about MEA Recordings, approximation of distinct intramural CVs, pseudo ECG calculations. (PDF) [file pone.0182979.s001.pdf]

S1 Text: Supplemental Methods

Modeling hypothermia induced effects for the heterogeneous ventricular tissue from cellular level to the impact on the ECG

[1]Roland Kienast, [1]Michael Handler, [1]Markus Stöger, [1,3]Daniel Baumgarten, [1]Friedrich Hanser, [1,2]Christian Baumgartner

[1] Institute of Electrical and Biomedical Engineering, UMIT – University for Health Sciences, Medical Informatics and Technology, A-6060 Hall in Tyrol, Austria

[2] Institute of Health Care Engineering with European Testing Center of Medical Devices, Graz University of Technology, A-8010 Graz, Austria

[3]Institute of Biomedical Engineering and Informatics, Technische Universität Ilmenau, D-98693 Ilmenau, Germany

Content

Supplemental Methods.......2

Experimental Setup.......2

MEA recordings.......2

Design of the 3D ventricular wedge model.......2

Approximation of distinct intramural CVs.......2

Validation.......3

Pseudo ECG (pECG) calculations.......3

References.......4

Supplemental Methods

Experimental Setup

Cell cultivation

We analyzed surface field potentials (FPs) recorded from n=18 primary cultures of chicken cardiomyocytes. As recently described in [1,2] the hearts of twelve-day-old chicken embryos were extracted. To exclude atrial myocardial cells from the cell preparation, ventricles were isolated from the atrium, minced and digested with 0.05% trypsin for separation. One drop of the obtained cell suspension was plated onto the fibronectin-coated microelectrode array (MEA), covering the electrode grid located in the center of a petri-dish. One to two minutes thereafter the petri-dish was carefully filled with 1.5ml of culture medium. One ml of medium was renewed daily. After three to four days of incubation, the cells formed a firmly attached and spontaneous beating monolayer, covering the whole electrode area used for MEA recordings. After the registration of FPs under hypothermal conditions, each cellular preparation was ineligible for further measurements and discarded.

MEA recordings

We used planar MEAs with integrated culture dish and three different electrode configurations, and a suitable recording system (Multi Channel Systems, Reutlingen, Germany) for extracellular recordings as described in [1]. The system was modified to operate in an adjustable temperature range from T = 10°C to T = 40°C to enable measurements under hypothermal conditions [2]. All measurements started at T = 37°C. The temperature was monotonically reduced in steps of 2°C every 5±1 minutes and field potentials were registered until spontaneous excitation collapsed. Data was registered simultaneously from all 60 channels with a sampling frequency of 20kHz each and a bandwidth of 1Hz to 3kHz. The registered signals were analyzed offline with an in-house developed software tool based on MATLAB (The Mathworks, Natick, MA, USA) and relevant intrinsic features such as the field potential rise time (FP_{rise}) and duration (FP_{dur}), the height of negative peak of the FP (FP_{min}) and the conduction velocity (CV) were extracted.

Design of the 3D ventricular wedge model

The monodomain equation was used for modeling the electrical excitation to determine the electrical propagation through the virtual ventricular tissue block (see Equation 1):

$$\frac{\partial V}{\partial t} = \nabla \cdot (D \nabla V) - \frac{I_{ion} + I_s}{C_m} \tag{1}$$

where V is the transmembrane potential, C_m is the membrane capacity, I_{ion} is the sum of all membrane currents, I_s the externally applied stimulus and D is a tensor, describing the anisotropic diffusion of the tissue in x, y and z direction (longitudinal, transversal and transmural).

Approximation of distinct intramural CVs

The number and conductivity of gap junctions is attributable to the intercellular resistance R_i and has an important impact on the intercellular electrical coupling. A reduction of the Cx43 in the epicardial cell layer therefore increases the epicardial transmural resistance compared to the M and endocardial layer [3]. This characteristic directly affects CV [4–6]. In computer models, intercellular resistance R_i is a parameter of the diffusion coefficient D, defined by

$$D = \frac{1}{S_v R_i C_m / A} \qquad (2)$$

where S_v is the surface to volume ratio, R_i the resistivity of the intracellular space (cytoplasm), C_m the membrane capacity and A the surface area. The intracellular space (cytoplasm) of myocardial cells is connected via gap junctions enabling cardiac excitation propagation. Therefore R_i combines the cytoplasmic (R_c) and gap junctional resistance (R_{gj}) [7] and can be expressed as $R_i = R_c + R_{gj}$.

Validation

Pseudo ECG (pECG) calculations

A pECG can be described as a bipolar measurement of the electric field of a myocardial wedge preparation placed in a conductive solution where the electrodes are commonly placed at a distance of 5 to 15mm from the epicardial and endocardial surface and it is commonly used in in-vitro studies. The acquired signals are similar to body surface ECGs [8]. For simulation of the pECGs, unipolar ECGs for a virtual electrode were calculated based on the transmembrane potential V_m in a domain B (tissue), utilizing the similar lead field concept as described in [9,10] using the following equation:

$$ECG_{el} = \int_B \frac{D \nabla V_m \cdot (\vec{r} - \vec{r}')}{|\vec{r} - \vec{r}'|^3} d^3 r' \qquad (3)$$

where D is the anisotropy diffusion tensor and $\vec{r}$ is the vector of the recording electrode and $\vec{r}'$ a vector to a point in the tissue. To calculate the bipolar pECG between two given electrodes $el1$ and $el2$ the bipolar ECG is defined as:

$$pECG_{bi} = ECG_{el2} - ECG_{el1} \qquad (4)$$

Combining equations 3 and 4 becomes

$$pECG_{bi} = \int_B D \nabla V_m \cdot \nabla \left(\frac{1}{r_1} - \frac{1}{r_2} \right) d^3 r' \qquad (5)$$

where r_1 and r_2 are the norms of the vectors from the recording electrodes to a point in tissue, respectively.

References

1. Kienast R, Stöger M, Handler M, Hanser F, Baumgartner C. Alterations of field potentials in isotropic cardiomyocyte cell layers induced by multiple endogenous pacemakers under normal and hypothermal conditions. Am J Physiol Heart Circ Physiol. 2014;307(7):H1013-23. doi: 10.1152/ajpheart.00097.2014. PubMed PMID: 25085965. eng.
2. Kienast R, Handler M, Stöger M, Fischer G, Hanser F, Baumgartner C. A system for analysing thermally-induced effects of propagation direction dependent features in field potentials of cardiomyocyte monolayers using multi-electrode arrays. In: Kennedy E, Macfarlane P., editors. Electrocardiology 2013: 40th International Congress on Electrocardiology.: Veda Publishing House. p. 159–64.
3. Yan GX, Shimizu W, Antzelevitch C. Characteristics and distribution of M cells in arterially perfused canine left ventricular wedge preparations. Circulation. 1998;98(18):1921–7. PubMed PMID: 9799214. eng.
4. Valderrabano M. Influence of anisotropic conduction properties in the propagation of the cardiac action potential. Prog Biophys Mol Biol. 2007;94(1-2):144–68. doi: 10.1016/j.pbiomolbio.2007.03.014.
5. Rohr S. Role of gap junctions in the propagation of the cardiac action potential. Cardiovasc Res. 2004;62(2):309–22. doi: 10.1016/j.cardiores.2003.11.035. PubMed PMID: 15094351. eng.
6. Kléber AG, Rudy Y. Basic mechanisms of cardiac impulse propagation and associated arrhythmias. Physiol Rev. 2004;84(2):431–88. doi: 10.1152/physrev.00025.2003. PubMed PMID: 15044680. eng.
7. Cabo C, Boyden PA. Extracellular space attenuates the effect of gap junctional remodeling on wave propagation: a computational study. Biophys J. 2009;96(8):3092–101. doi: 10.1016/j.bpj.2009.01.014. PubMed PMID: 19383455. eng.
8. Patel C, Burke JF, Patel H, Gupta P, Kowey PR, Antzelevitch C, et al. Is there a significant transmural gradient in repolarization time in the intact heart? Cellular basis of the T wave: a century of controversy. Circ Arrhythm Electrophysiol. 2009;2(1):80–8. doi: 10.1161/CIRCEP.108.791830. PubMed PMID: 19808446. eng.
9. Ten Tusscher, Kirsten H W J, Bernus O, Hren R, Panfilov AV. Comparison of electrophysiological models for human ventricular cells and tissues. Prog Biophys Mol Biol. 2006;90(1-3):326–45. doi: 10.1016/j.pbiomolbio.2005.05.015. PubMed PMID: 16002127. eng.
10. Weinberg S, Iravanian S, Tung L. Representation of collective electrical behavior of cardiac cell sheets. Biophys J. 2008;95(3):1138–50. doi: 10.1529/biophysj.107.128207. PubMed PMID: 18469085. eng.
