# Supplementary material for: Modeling hypothermia induced effects for the heterogeneous ventricular tissue from cellular level to the impact on the ECG
Source: PLoS One. 2017 Aug 16;12(8):e0182979. doi: 10.1371/journal.pone.0182979 (PMC5558962; doi:10.1371/journal.pone.0182979)
Supplement: S2 Text — (PDF) [file pone.0182979.s002.pdf]

S2 TEXT: Q10 VALUES

Modeling hypothermia induced effects for the heterogeneous ventricular tissue from cellular level to the impact on the ECG

[1]Roland Kienast, [1]Michael Handler, [1]Markus Stöger, [1,3]Daniel Baumgarten, [1]Friedrich Hanser, [1,2]Christian Baumgartner

[1] Institute of Electrical and Biomedical Engineering, UMIT – University for Health Sciences, Medical Informatics and Technology, A-6060 Hall in Tyrol, Austria

[2] Institute of Health Care Engineering with European Testing Center of Medical Devices, Graz University of Technology, A-8010 Graz, Austria

[3]Institute of Biomedical Engineering and Informatics, Technische Universität Ilmenau, D-98693 Ilmenau, Germany

Content

Q10 values extracted from literature 2

- APD 2
- V_{max} 2
- CL 3
- CV 3

References 4

Q10 values extracted from literature

This supplemental includes estimated Q_{10} values based on literature where the Q10 values (APD, V_{max}, CL, CV) are calculated based on two values for different temperatures, respectively, using

$$Q_{10} = \left(\frac{X_{high}}{X_{low}} \right)^{\left(\frac{10}{T_{high} - T_{low}} \right)} \quad (5)$$

where X_{high} denotes the value of the investigated feature at higher temperature T_{high} and X_{low} the value at the lower temperature T_{low}. Thereby, the two values at high and low temperature are extracted from textual description or estimated based on available graphics in the respective literature. Table I summarizes the results for APD, Table II for V_{max}, Table III for CL and Table IV for CV.

APD

Table I: Estimated Q_{10} values for APD based on literature

Q_{10}	Measure	Species	Tissue	Temperature range	Reference
0.66	APD90	guinea-pig	ventricular papillary muscle	37-27	[1]
0.83	APD95	rabbit	left ventricle	33-23	[2]
0.53	APD90	rabbit	isolated papillary muscles	37-27	[3]
0.44	APD90	guinea-pig	ventricular papillary muscle	37-27	[4]
0.48	APD90	guinea-pig	ventricular and atrial cells	35-25	[5]
0.75	APD90	pig	ventricular septum	37-32	[6]
0.61	APD70	rabbit	ventricle	37-17	[7]
0.58	APD	dog	left ventricle	36-32	[8]
0.55	APD90	rats	papillary muscles	37-10	[9]

V_{max}

Table II: Estimated Q_{10} values for V_{max} based on literature

Q_{10}	Species	Tissue	Temperature range	Reference
1.56	guinea-pig	ventricular papillary muscle	37-27	[1]
1.51	chicken	right ventricle	37-20	[10]
1.59	rabbit	left ventricle	33-23	[2]
1.65	rabbit	isolated papillary muscles	37-27	[3]
1.4	guinea-pig	ventricular papillary muscle	37-27	[4]
2.35	guinea-pig	ventricular cells	35-25	[5]

The obtained Q_{10} value for V_{max} of 1.68 used in the model was estimated by calculating the mean value of all listed Q10 values in table II.

CL

In literature, the information about heart rate is usually indicated by beats per minute (ppm). To convert values from ppm to CL following formula was used

$$CL = \frac{60}{ppm}$$

where CL denotes the value for the cycle length in seconds and ppm the value for the heart rate in beats per minute.

Table III: Estimated Q_{10} values for CL based on literature

Q_{10ppm}	Q_{10CL}	Species	Temperature range	Reference
3	0.35	rat	34-23	[11]
2	0.5	pig	37-31	[12]
3.6	0.28	dog	40-20.5	[13]

CV

Table IV: Estimated Q_{10} values for CV based on literature

Q_{10}	Species	Tissue	Temperature range	Reference
1.5	chicken	right ventricle	37-20	[10]
1.39	rabbit	ventricle	37-27	[7]
1.47	rabbit	ventricle	37-27	[14]
1.59	dog	left ventricle	36-26	[15]

References

1. Melnikov AL, Lathrop DA, Helgesen KG. Diazepam-induced Ca(2+)-channel blockade reduces hypothermia-induced electromechanical changes in isolated guinea pig ventricular muscle. Eur J Anaesthesiol. 1998;15(1):96–102. PubMed PMID: 9522148. eng.
2. Samson JP, Reisin I, Ruiz-Ceretti E, Schanne OF. Effects of low termperature on intracellular ionic concentrations and transmembrane potential in isolated rabbit hearts. J Mol Cell Cardiol. 1977;9(1):39–50. PubMed PMID: 845974. eng.
3. Fedorov VV, Li L, Glukhov A, Shishkina I, Aliev RR, Mikheeva T, et al. Hibernator Citellus undulatus maintains safe cardiac conduction and is protected against tachyarrhythmias during extreme hypothermia: possible role of Cx43 and Cx45 up-regulation. Heart Rhythm. 2005;2(9):966–75. doi: 10.1016/j.hrthm.2005.06.012. PubMed PMID: 16171752. eng.
4. Bjørnstad H, Tande PM, Lathrop DA, Refsum H. Effects of temperature on cycle length dependent changes and restitution of action potential duration in guinea pig ventricular muscle. Cardiovasc Res. 1993;27(6):946–50. PubMed PMID: 8221783. eng.
5. Hume JR, Uehara A. Ionic basis of the different action potential configurations of single guinea-pig atrial and ventricular myocytes. J Physiol. 1985;368:525–44. PubMed PMID: 2416918. eng.
6. Roscher R, Arlock P, Sjöberg T, Steen S. Effects of dopamine on porcine myocardial action potentials and contractions at 37 degrees C and 32 degrees C. Acta Anaesthesiol Scand. 2001;45(4):421–6. PubMed PMID: 11300379. eng.
7. Egorov YV, Glukhov AV, Efimov IR, Rosenshtraukh LV. Hypothermia-induced spatially discordant action potential duration alternans and arrhythmogenesis in nonhibernating versus hibernating mammals. Am J Physiol Heart Circ Physiol. 2012;303(8):H1035-46. doi: 10.1152/ajpheart.00786.2011. PubMed PMID: 22886418. eng.
8. Piktel JS, Rosenbaum DS, Wilson LD. Mild hypothermia decreases arrhythmia susceptibility in a canine model of global myocardial ischemia*. Crit Care Med. 2012;40(11):2954–9. doi: 10.1097/CCM.0b013e31825fd39d. PubMed PMID: 22890250. eng.
9. Wang S-Q, Cao H-M, Zhou Z-Q. Temperature dependence of the myocardial excitability of ground squirrel and rat. J Therm Biol. 1997;22(3):195–9. doi: 10.1016/S0306-4565(97)00010-7.
10. Hirota A, Fujii S, Sakai T, Kamino K. Temperature dependence of spontaneous electrical activity in early embryonic heart monitored optically with a potential-sensitive dye. Jpn J Physiol. 1983;33(1):85–100. PubMed PMID: 6855032. eng.
11. Harary I, Farley B. In vitro studies on single beating rat heart cells. II. Intercellular communication. Exp Cell Res. 1963;29:466–74. PubMed PMID: 13952709. eng.
12. Weisser J, Martin J, Bisping E, Maier LS, Beyersdorf F, Hasenfuss G, et al. Influence of mild hypothermia on myocardial contractility and circulatory function. Basic Res Cardiol. 2001;96(2):198–205. PubMed PMID: 11327339. eng.
13. Berne RM. Myocardial Function in Severe Hypothermia. Circ Res. 1954;2(1):90–5. doi: 10.1161/01.RES.2.1.90.
14. Fedorov VV, Glukhov AV, Sudharshan S, Egorov Y, Rosenshtraukh LV, Efimov IR. Electrophysiological mechanisms of antiarrhythmic protection during hypothermia in winter hibernating versus nonhibernating mammals. Heart Rhythm. 2008;5(11):1587–96. doi: 10.1016/j.hrthm.2008.08.030. PubMed PMID: 18984537. eng.
15. Piktel JS, Jeyaraj D, Said TH, Rosenbaum DS, Wilson LD. Enhanced dispersion of repolarization explains increased arrhythmogenesis in severe versus therapeutic hypothermia. Circ Arrhythm Electrophysiol. 2011;4(1):79–86. doi: 10.1161/CIRCEP.110.958355. PubMed PMID: 21163888. eng.
