# Supplementary material for: Modeling hypothermia induced effects for the heterogeneous ventricular tissue from cellular level to the impact on the ECG
Source: PLoS One. 2017 Aug 16;12(8):e0182979. doi: 10.1371/journal.pone.0182979 (PMC5558962; doi:10.1371/journal.pone.0182979)
Supplement: S1 Fig — (PDF) [file pone.0182979.s003.pdf]

# S1 Fig.

**Modeling hypothermia induced effects for the heterogeneous ventricular tissue from cellular level to the impact on the ECG**

[1]Roland Kienast, [1]Michael Handler, [1]Markus Stöger, [1,3]Daniel Baumgarten, [1]Friedrich Hanser, [1,2]Christian Baumgartner

[1] Institute of Electrical and Biomedical Engineering, UMIT – University for Health Sciences, Medical Informatics and Technology, A-6060 Hall in Tyrol, Austria

[2] Institute of Health Care Engineering with European Testing Center of Medical Devices, Graz University of Technology, A-8010 Graz, Austria

[3]Institute of Biomedical Engineering and Informatics, Technische Universität Ilmenau, D-98693 Ilmenau, Germany

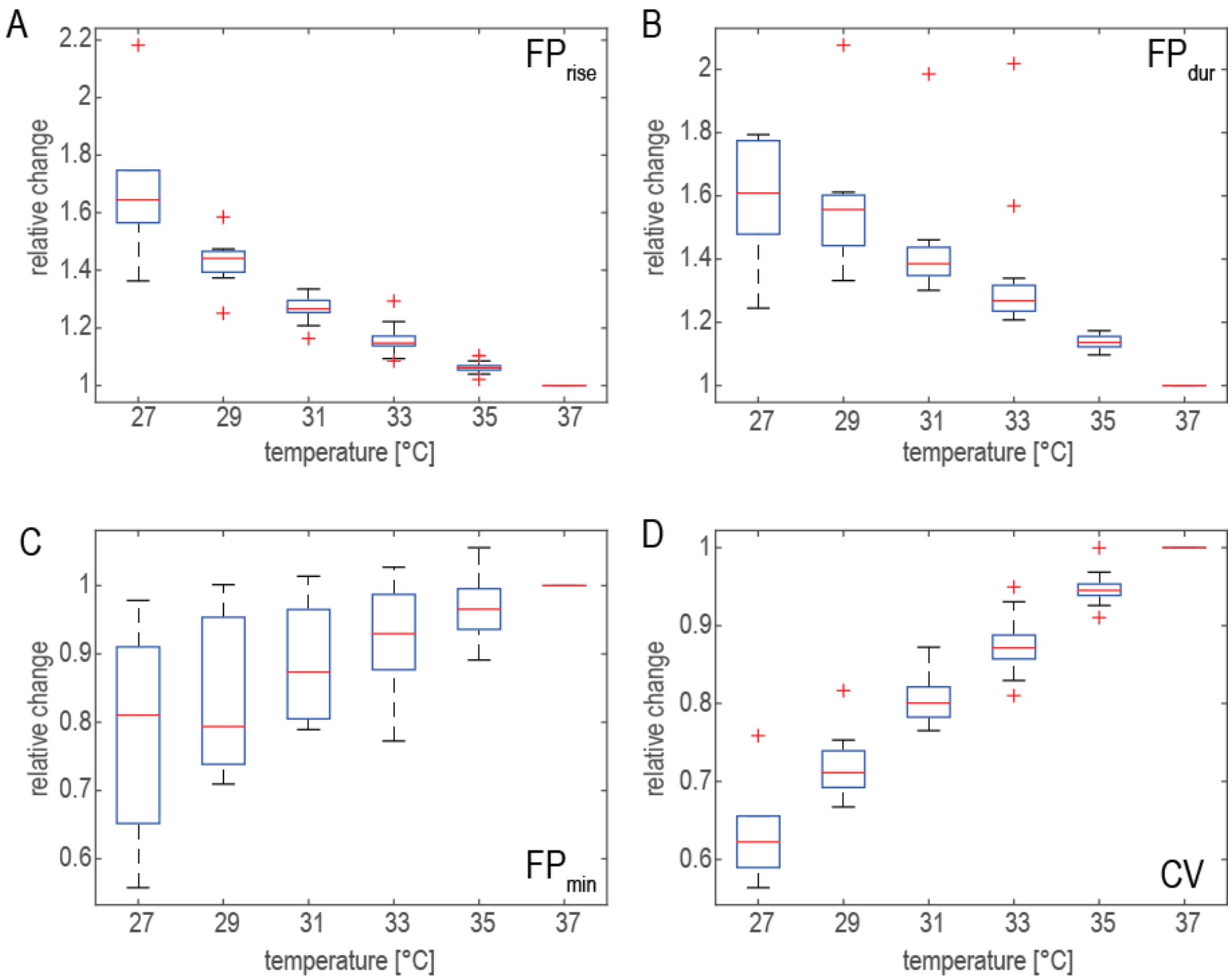


Fig S1: Boxplots of the relative changes of FP parameters ($FP_{rise}$,$FP_{dur}$,$FP_{min}$) and CV referred to a temperature of 37°C for n=18 primary cultures of chicken cardiomyocytes.
