# Supplementary material for: Modeling hypothermia induced effects for the heterogeneous ventricular tissue from cellular level to the impact on the ECG
Source: PLoS One. 2017 Aug 16;12(8):e0182979. doi: 10.1371/journal.pone.0182979 (PMC5558962; doi:10.1371/journal.pone.0182979)
Supplement: S2 Fig — (PDF) [file pone.0182979.s004.pdf]

S2 FIG.

Modeling hypothermia induced effects for the heterogeneous ventricular tissue from cellular level to the impact on the ECG

[1]Roland Kienast, [1]Michael Handler, [1]Markus Stöger, [1,3]Daniel Baumgarten, [1]Friedrich Hanser, [1,2]Christian Baumgartner

[1] Institute of Electrical and Biomedical Engineering, UMIT – University for Health Sciences, Medical Informatics and Technology, A-6060 Hall in Tyrol, Austria

[2] Institute of Health Care Engineering with European Testing Center of Medical Devices, Graz University of Technology, A-8010 Graz, Austria

[3]Institute of Biomedical Engineering and Informatics, Technische Universität Ilmenau, D-98693 Ilmenau, Germany

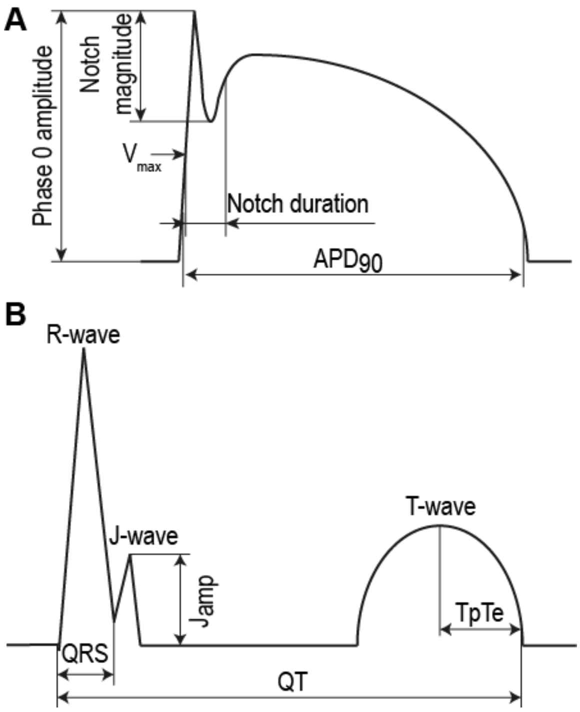

Fig S2: Schematic illustration of (A) a single AP complex and the determined parameters and (B) of a single pECG complex and the determined ECG parameters.
