# Supplementary material for: Modeling hypothermia induced effects for the heterogeneous ventricular tissue from cellular level to the impact on the ECG
Source: PLoS One. 2017 Aug 16;12(8):e0182979. doi: 10.1371/journal.pone.0182979 (PMC5558962; doi:10.1371/journal.pone.0182979)
Supplement: S3 Fig — (PDF) [file pone.0182979.s005.pdf]

S3 Fig.

Modeling hypothermia induced effects for the heterogeneous ventricular tissue from cellular level to the impact on the ECG

[1]Roland Kienast, [1]Michael Handler, [1]Markus Stöger, [1,3]Daniel Baumgarten, [1]Friedrich Hanser, [1,2]Christian Baumgartner

[1] Institute of Electrical and Biomedical Engineering, UMIT – University for Health Sciences, Medical Informatics and Technology, A-6060 Hall in Tyrol, Austria

[2] Institute of Health Care Engineering with European Testing Center of Medical Devices, Graz University of Technology, A-8010 Graz, Austria

[3]Institute of Biomedical Engineering and Informatics, Technische Universität Ilmenau, D-98693, Ilmenau, Germany

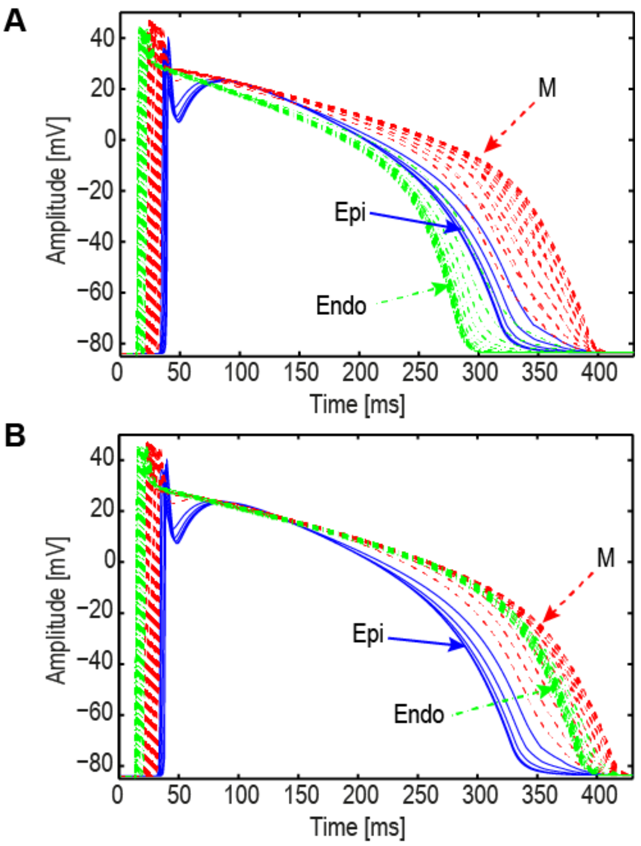

Fig S3: (A) Simulated transmural APs without APD correction where endocardial cells show the shortest APD. In normal left ventricular tissue, however, epicardial cells generate the shortest APDs followed by the endocardial cells and the M cells. (B) Simulated transmural APs with APD correction to maintain the physiological APD sequence (epi-endo-M). Intracellular electrically-coupled cells tend to average distinct myocardial action potentials. This averaging effect is obvious between the different cell types with and without APD correction.
