# Supplementary material for: Modeling hypothermia induced effects for the heterogeneous ventricular tissue from cellular level to the impact on the ECG
Source: PLoS One. 2017 Aug 16;12(8):e0182979. doi: 10.1371/journal.pone.0182979 (PMC5558962; doi:10.1371/journal.pone.0182979)
Supplement: S1 Table — Detailed information about all absolute FP values and CV estimated at different temperatures for n = 18 cell layer. (PDF) [file pone.0182979.s006.pdf]

S1 Table

Modeling hypothermia induced effects for the heterogeneous ventricular tissue from cellular level to the impact on the ECG

[1]Roland Kienast, [1]Michael Handler, [1]Markus Stöger, [1,3]Daniel Baumgarten, [1]Friedrich Hanser, [1,2]Christian Baumgartner

[1] Institute of Electrical and Biomedical Engineering, UMIT – University for Health Sciences Medical Informatics and Technology, A-6060 Hall in Tyrol, Austria
[2]Institute of Health Care Engineering with European Testing Center of Medical Devices, Graz University of Technology, A-8010 Graz, Austria
[3]Institute of Biomedical Engineering and Informatics, Technische Universität Ilmenau, D-98693 Ilmenau, Germany

Table S1: Detailed information about all absolute values estimated at different temperatures for n=18 cell layer.

		Fprise ms			Fpdur ms			Fpmin mV			CV cm/s		
Cell Culture	Temp. [°C]	Group Count	Median	MAD	Group Count	Median	MAD	Group Count	Median	MAD	Group Count	Median	MAD
1	31	2381	0,163	0,094	1779	344	82	2381	-1,17	0,33	1662	14,8	1,3
	33	2486	0,146	0,110	1897	301	73	2487	-1,35	0,37	1724	17,1	1,6
	35	2092	0,132	0,057	1364	312	141	2092	-1,33	0,35	1464	18,7	1,7
	37	8012	0,126	0,039	4980	276	96	8012	-1,20	0,33	5721	18,7	1,8
2	31	36736	0,126	0,023	36423	311	10	36736	-1,55	0,45	16992	22,7	2,1
	33	5752	0,117	0,025	5720	272	8	5752	-1,65	0,44	2664	25,0	2,0
	35	12650	0,109	0,023	12603	243	5	12650	-1,69	0,45	5856	26,5	2,0
	37	16551	0,102	0,020	16341	214	4	16551	-1,74	0,48	7656	27,8	2,1
3	21	893	0,253	0,053	728	577	189	893	-1,66	0,59	380	14,9	0,7
	23	2115	0,223	0,047	1803	510	145	2115	-2,20	0,57	900	15,4	1,3
	25	3697	0,199	0,060	3229	455	113	3697	-2,38	0,65	1524	17,1	1,5
	27	2269	0,178	0,046	2068	414	52	2269	-2,66	0,70	921	19,4	1,7
	29	3400	0,162	0,035	3197	382	44	3400	-2,75	0,77	1346	22,3	1,9
	31	4671	0,146	0,033	4450	362	34	4671	-2,65	0,78	1948	24,0	2,0
	33	5271	0,142	0,030	5090	339	29	5271	-2,92	0,76	2260	23,7	2,3
	35	5928	0,136	0,029	5735	312	25	5928	-2,95	0,76	2536	24,5	2,4
	37	22502	0,130	0,030	21295	284	23	22502	-2,90	0,75	9564	25,2	2,4

4	21	878	0,339	0,047	422	942	319	878	-0,37	0,17	1455	15,0	0,9
	23	2380	0,261	0,040	1153	898	363	2380	-0,39	0,18	1466	17,8	1,1
	25	2437	0,214	0,032	1327	663	364	2437	-0,48	0,19	1547	20,5	1,4
	27	2503	0,183	0,032	1670	494	349	2503	-0,52	0,20	3040	22,6	1,7
	29	4975	0,164	0,039	3783	339	224	4975	-0,56	0,19	4146	25,0	2,0
	31	6834	0,146	0,030	5554	306	82	6834	-0,57	0,19	6522	26,9	2,1
	33	10720	0,135	0,025	9268	278	30	10720	-0,56	0,19	7510	28,9	2,3
	35	12340	0,125	0,023	11334	242	19	12340	-0,57	0,20	10276	30,8	2,3
	37	16848	0,117	0,022	15562	211	15	16848	-0,56	0,19	7197	30,8	2,2
5	21	629	0,338	0,057	449	815	253	629	-1,00	0,17	720	15,5	0,6
	23	1272	0,240	0,041	1023	502	235	1272	-1,33	0,24	1680	18,5	0,7
	25	2968	0,193	0,032	2614	421	200	2968	-1,52	0,28	3300	21,4	0,9
	27	5830	0,164	0,027	5448	378	77	5830	-1,66	0,31	3300	23,9	1,3
	29	5830	0,144	0,024	5592	343	19	5830	-1,76	0,36	3480	26,8	1,4
	31	6148	0,126	0,018	6038	317	12	6148	-1,79	0,38	2670	29,4	1,5
	33	4717	0,115	0,016	4666	289	10	4717	-1,82	0,39	3510	32,2	1,6
	35	6201	0,107	0,015	6158	258	6	6201	-1,82	0,41	5550	34,0	1,7
	37	9804	0,100	0,014	9717	225	8	9804	-1,78	0,39	1950	33,7	1,7
6	29	2128	0,160	0,024	1659	608	350	2128	-1,48	0,33	1890	21,1	1,4
	31	3024	0,144	0,022	2664	382	362	3024	-1,60	0,34	4130	22,2	1,6
	33	6611	0,137	0,020	6043	266	179	6611	-1,72	0,37	6300	24,2	1,6
	35	10080	0,124	0,017	9799	240	23	10080	-1,81	0,40	19565	26,7	1,6
	37	31304	0,112	0,014	30532	208	9	31304	-1,85	0,42	1321	15,7	1,4
7	31	2538	0,212	0,106	1707	860	408	2538	-0,21	0,06	1516	17,1	1,5
	33	3926	0,192	0,091	2960	497	422	3926	-0,23	0,08	1869	18,7	1,7
	35	4520	0,176	0,091	3462	425	358	4520	-0,23	0,08	3926	19,9	1,8
	37	8402	0,164	0,082	6264	392	310	8402	-0,22	0,08	750	15,2	1,0

8	27	1375	0,210	0,029	1146	332	324	1375	-1,49	0,37	2400	18,0	1,3
	29	4400	0,173	0,024	4165	306	129	4400	-1,65	0,44	3804	20,5	1,5
	31	6983	0,154	0,024	6808	281	25	6983	-1,86	0,47	4260	22,9	1,7
	33	7810	0,142	0,022	7694	256	36	7810	-2,11	0,49	4680	25,6	1,8
	35	8580	0,127	0,019	8489	233	9	8580	-2,08	0,50	5430	27,0	1,9
	37	9955	0,121	0,018	9769	212	8	9955	-2,13	0,49	1980	17,6	1,3
9	29	168	0,256	0,143	36	1048	459	168	-0,18	0,06	280	17,6	2,0
	31	448	0,227	0,119	167	978	414	448	-0,18	0,07	557	19,2	2,2
	33	901	0,207	0,121	401	990	432	901	-0,19	0,07	3900	20,9	2,5
	35	6198	0,194	0,173	4661	268	408	6198	-0,20	0,08	6443	21,4	2,7
	37	10558	0,188	0,114	8875	241	169	10558	-0,24	0,08	490	18,9	2,3
10	25	2164	0,254	0,048	2040	479	63	2164	-1,02	0,31	1053	18,5	1,4
	27	1800	0,214	0,052	1628	435	273	1800	-1,19	0,37	1290	21,7	1,7
	29	2107	0,182	0,056	2062	384	203	2107	-1,34	0,41	1735	24,2	2,0
	31	2837	0,161	0,051	2830	345	25	2837	-1,47	0,45	2370	26,1	2,3
	33	3871	0,153	0,055	3764	311	34	3871	-1,61	0,49	3450	28,0	2,3
	35	5635	0,145	0,049	5129	275	25	5635	-1,73	0,53	5130	29,8	2,4
	37	8379	0,135	0,048	6689	234	17	8379	-1,82	0,56	1031	18,9	1,4
11	29	5218	0,201	0,065	4456	294	319	5218	-1,09	0,34	5495	20,3	1,6
	31	8792	0,162	0,027	7798	256	139	8792	-1,53	0,43	5810	22,4	1,7
	33	9296	0,145	0,024	8490	234	76	9296	-1,66	0,48	9764	25,1	1,7
	35	15619	0,123	0,017	14411	203	19	15619	-1,80	0,51	19209	26,2	1,9
	37	30729	0,116	0,016	28618	174	13	30729	-1,82	0,53	5495	21,6	1,6
12	33	6991	0,208	0,050	5784	191	76	6991	-1,37	0,39	3576	25,3	2,7
	35	6537	0,178	0,045	6053	167	44	6537	-1,61	0,43	6731	27,4	2,9
	37	12254	0,151	0,038	9812	154	97	12254	-1,75	0,46	7824	27,0	2,7
13	31	7000	0,163	0,027	6054	301	235	7000	-1,65	0,47	5314	23,1	1,7
	33	8509	0,137	0,022	7432	265	109	8509	-1,81	0,53	6300	25,0	1,8
	35	10080	0,125	0,019	8939	234	46	10080	-2,01	0,55	7595	26,8	1,9
	37	12151	0,115	0,017	11063	205	24	12151	-2,12	0,58	4830	22,5	1,7
14	31	2681	0,249	0,074	2641	203	44	2681	-1,27	0,44	3094	22,4	2,6
	33	5824	0,212	0,043	5750	186	35	5824	-1,61	0,45	1860	25,6	3,3
	35	3712	0,176	0,050	3047	174	24	3712	-1,85	0,47	6669	27,8	3,2
	37	12957	0,165	0,041	11795	154	21	12957	-2,07	0,54	3729	27,9	4,0

15	29	6210	0,225	0,040	5475	404	20	6210	-0,53	0,12	4536	20,9	2,3
	31	5832	0,203	0,037	5425	371	11	5832	-0,53	0,14	7095	22,2	1,7
	33	8910	0,191	0,053	8660	335	8	8910	-0,59	0,14	7783	24,2	1,9
	35	9774	0,175	0,050	9564	296	5	9774	-0,61	0,14	48546	25,6	2,1
	37	61830	0,164	0,046	59593	259	4	61830	-0,64	0,15	3318	18,5	1,9
16	29	3190	0,197	0,029	2019	434	20	3190	-2,04	0,51	2343	23,9	1,9
	31	3905	0,178	0,026	2887	404	21	3905	-2,18	0,55	3762	25,7	2,1
	33	6270	0,161	0,023	5186	369	9	6270	-2,37	0,58	4257	27,5	2,3
	35	7095	0,149	0,021	6282	333	8	7095	-2,54	0,60	27670	29,0	2,5
	37	46644	0,140	0,022	43964	290	8	46645	-2,71	0,62	165	13,2	1,5
17	29	4551	0,303	0,077	4094	353	20	4551	-0,55	0,29	4800	27,6	3,8
	31	6840	0,206	0,046	6592	332	19	6840	-0,78	0,41	5465	29,9	3,3
	33	8051	0,163	0,039	7858	314	12	8051	-1,08	0,44	6431	35,0	4,1
	35	9295	0,144	0,041	9113	285	14	9295	-1,17	0,51	28488	38,2	4,8
	37	40632	0,146	0,033	39456	253	8	40633	-1,37	0,59	2431	20,5	3,3
18	25	2777	1,246	3,004	165	743	372	2777	-0,14	0,14	2653	15,0	2,6
	27	3359	0,492	2,033	678	374	430	3359	-0,40	0,31	4565	20,2	40,6
	29	6563	0,256	0,158	5095	293	123	6566	-1,30	0,46	5019	22,8	2,1
	31	6956	0,220	0,074	5824	292	100	6956	-1,45	0,47	7945	24,8	2,2
	33	10511	0,198	0,043	9279	272	41	10511	-1,59	0,50	8273	26,3	2,5
	35	10748	0,184	0,039	9835	251	31	10748	-1,68	0,54	29653	28,9	3,4
	37	41108	0,172	0,051	35987	223	26	41114	-1,83	0,60	631	6,4	1,9
